# Clinical value and expression of Homer 1, homocysteine, S-adenosyl-l-homocysteine, fibroblast growth factors 23 in coronary heart disease

**DOI:** 10.1186/s12872-022-02554-4

**Published:** 2022-05-12

**Authors:** Zhixin Zhang, Lin Wang, Yu Zhan, Cui Xie, Yang Xiang, Dan Chen, You Wu

**Affiliations:** Department of Cardiology, Renmin Hospital, Hubei University of Medicine, No. 39 Chaoyang Middle Road, Shiyan, 442000 Hubei China

**Keywords:** Homer 1, S-adenosyl-l-homocysteine, Homocysteine, Fibroblast growth factors 23, Coronary heart disease

## Abstract

**Background:**

This study aimed to explore clinical value and expression of Homer 1, S-adenosyl-l-homocysteine (SAH), homocysteine (Hcy), fibroblast growth factors (FGF) 23 in coronary heart disease (CHD).

**Methods:**

From March 2020 to April 2021, a total of 137 patients with CHD and 138 healthy subjects who came to our hospital for physical examination and had no cardiovascular disease were retrospectively enrolled, and they were assigned to the CHD group and the control group, respectively. Patients in the CHD group were divided into stable angina pectoris (SAP) group (n = 48), unstable angina pectoris (UAP) group (n = 46), and acute myocardial infarction (AMI) group (n = 43) according to clinical characteristics for subgroup analysis. The degree of coronary artery stenosis was assessed by Gensini score, which is a reliable assessment tool for the severity of coronary artery disease. The levels of Homer 1, SAH, Hcy, and FGF 23 were tested and compared. Spearman correlation analysis was used to analyze the correlation between serum Homer1, SAH, Hcy, FGF23 levels and Gensini score, and multivariate unconditional Logistic regression was used to analyze the risk factors of coronary heart disease.

**Results:**

Demographic characteristics of each group were comparable (*P* > 0.05). The body mass index (BMI), total cholesterol (TC), low-density lipoprotein cholesterol (LDL-C), triglyceride (TG), and glucose levels of the SAP group, UAP group and AMI group were significantly higher than those of the control group, and the number of patients with smoking, alcohol consumption, hypertension, and diabetes history was significantly more than that of the control group, respectively (*P* < 0.05). The level of high-density lipoprotein cholesterol (HDL-C) of each subgroup was significantly lower than the control group (*P* < 0.05). The above indicators showed no significant difference among three subgroups (*P* > 0.05). Serum SAH, Hcy, Homer1 and FGF23 levels in each subgroup were significantly higher than those in control group (*P* < 0.05). And above indicators in SAP group and UAP group were significantly lower than those in AMI group (*P* < 0.05), and the levels of above indicators in SAP group were significantly lower than those in UAP group (*P* < 0.05). The results of Spearman correlation analysis showed that serum Homer1, FGF23, SAH, Hcy levels were positively correlated with Gensini score (r = 0.376, 0.623, 0.291, 0.372, all *P* < 0.01). Multivariate logistic regression analysis showed that smoking, hypertension, diabetes, alcohol consumption, obesity, HDL-C, FGF23, SAH, Hcy, Homer 1 were independent risk factors for coronary heart disease.

**Conclusion:**

The levels of FGF23, SAH, Hcy, and Homer1 tend to increase in patients with CHD compared with normal population, and the more severe the disease, the higher the levels, which has certain reference value for the clinical diagnosis of CHD and the evaluation and monitoring of the disease.

## Background

Coronary heart disease (CHD) is a common clinical chronic cardiovascular disease, which is mainly caused by atherosclerosis-induced arterial stenosis and hardening of the wall, resulting in insufficient blood supply to myocardial cells [1, 2]. It can induce other cardiovascular diseases, and the mortality and morbidity are both high, which seriously endangers the health of patients [1, 3]. It is of great clinical significance to timely discover the risk factors of coronary heart disease and adopt timely and effective prevention and treatment measures for patients with coronary heart disease, so as to avoid causing other cardiovascular diseases and improve cardiac function of patients. The risk factors of CHD include invariable factors (e.g. age, gender, etc.) and variable factors (e.g. dyslipidemia, hypertension, diabetes, smoking, etc.) [4].

Homocysteine (Hcy), an important intermediate product of methionine and cysteine metabolism, has certain toxic effects on vascular endothelial cells, causing endothelial damage and dysfunction, leading to proliferation of vascular smooth muscle cells, destruction of the balance between coagulation and fibrinolysis, and resulting in the body in a pre-thrombosis state [5]. Studies have shown that Hcy is closely related to CHD and can be used as an independent risk factor for the diagnosis of CHD [6, 7]. Homer protein family members are encoded by three genes (Homer1, 2 and 3), and the three subtypes are also called Vesl, PSD-Zip45, and Cupidin [8]. Homer is widely distributed in the heart and skeletal muscles. By participating in the regulation of the activity of a variety of calcium channel proteins in the cell, it regulates the intracellular calcium ion concentration, thereby playing an important role in cardiovascular [9]. S-adenosyl-l-homocysteine (SAH) is the sole metabolic precursor of Hcy in a reversible reaction catalyzed by SAH hydrolase [10], both of which are intermediate metabolites of methionine, and SAH is an effective inhibitor of DNA methyltransferase, which can initiate abnormal DNA methylation. A large sample cohort study suggested that higher level of FGF 23 was independently associated with greater risk of incident coronary heart disease [11].

However, there are few reports about whether Homer1, SAH, Hcy and FGF23 are involved in the occurrence and development of CHD. Therefore, this study aims to explore the expression and clinical value of Homer1, SAH, Hcy, and FGF23 in CHD.

## Materials and methods

### Clinical data

From March 2020 to April 2021, 137 cases of CHD patients and 138 cases of healthy population, who came to our hospital for physical examination and had no cardiovascular disease, were retrospectively enrolled in Renmin Hospital, Hubei University of Medicine, and they were assigned to the CHD group and the control group, respectively. The patients in CHD group were divided into three subgroups, namely, stable angina pectoris (SAP) group (n = 48), unstable angina pectoris (UAP) group (n = 46), and acute myocardial infarction (AMI) group (n = 43), according to clinical symptoms and results of coronary arteriography. The study protocol was approved by the Ethics Committee of Renmin Hospital, Hubei University of Medicine. The formulation of this study scheme was in accordance with the requirements of the Declaration of Helsinki of the World Medical Association. Written informed consent was obtained from all participants.

### Inclusion and exclusion criteria

The inclusion criteria are as follows: (1) The diagnosis met the diagnostic criteria of CHD in the 2012 American College of Cardiology Foundation/American Heart Association task force on practice guidelines, and the American College of Physicians, American Association for Thoracic Surgery, Preventive Cardiovascular Nurses Association, Society for Cardiovascular Angiography and Interventions, and Society of Thoracic Surgeons Guideline [12] and 2018 Chinese Guideline [13]. (2) Patients with normal liver, kidney and thyroid function. (3) Patients in good mental health and good compliance. (4) No heart coronary artery bypass grafting or percutaneous coronary intervention was performed within half a year. (5) Signed informed consent.

The exclusion criteria are as follows: (1) Patients with malignant tumors. (2) Patients with acute infection. (3) Patients complicated with hyperlipidemia, hypertension, diabetes, hyperuricemia or immune system diseases. (4) Critically ill patients. (5) Severely anemic patients. (6) Pregnant or lactating women.

## Methods

The clinical characteristics including gender, age, history of illness, body mass index (BMI), systolic blood pressure (SBP), diastolic blood pressure (DBP) were collected. After 8 h fasting, 5-8 mL of venous blood was collected by coagulation tube in the morning of the 2nd day. Total cholesterol (TC), high density lipoprotein cholesterol (HDL-C), low density lipoprotein cholesterol (LDL-C), triglyceride (TG), fasting blood-glucose (FBG) were tested using PPI automatic biochemical analyzer produced by Roche Diagnostics. SAH, Hcy, FGF23 levels were tested by enzyme-linked immunosorbent method ( Medicalsystem Biotechnology Co., Ltd., Ningbo, China). Quantitative PCR was used to detect the expression level of HOMER1 gene mRNA in peripheral blood leukocytes of all subjects. The PCR results were presented by cycle threshold (Ct) values. Each clinical sample was tested for 3 times, and its average value was taken as the final value. The mRNA relative expression level was calculated by 2^−△^Ct method.

The degree of coronary artery stenosis was assessed by Gensini score [14, 15]. According to the results of coronary angiography, the coronary arteries were divided into 14 segments, and the most severe stenosis of each segment was measured and scored. Three main parameters for each coronary lesion, namely, severity score, region multiplying factor and collateral adjustment factor were taken into consideration. A score of 1 represents 1–25% obstruction. In the process of reducing the diameter by 25–50%, 50–75%, 75–90%, 90–99% 99–100%, as the severity of the obstruction increases, the score doubles. That is, when the degree of obstruction is 99–100%, the score is 32 points. Weight coefficients of different vascular segments are as follows: left main coronary artery × 5, proximal anterior descending coronary artery × 2.5, middle anterior descending coronary artery × 1.5, distal anterior descending coronary artery × 1, proximal circumflex coronary artery × 2.5 (ostium × 3.5), distal anterior descending coronary artery, obtuse marginal, right coronary artery, posterior descending coronary artery, and the 1st diagonal are all × 1, 2nd diagonal and posterior coronary collateral × 0.5. The cumulative score of each segment lesion is the total score of the degree of coronary artery lesion of the patient.

Hypertension is defined as Elevated blood pressure measured at least 3 times on different days, with SBP ≥ 140 mmHg, and/or DBP ≥ 90 mmHg.

Obesity is defined as BMI ≥ 30 kg/m^2^ [16].

Diagnostic criteria of diabetes are as follows [17]: (1) Fasting plasma glucose ≥ 126 mg/dL (7.0 mmol/L). Fasting is defined as no caloric intake for at least 8 h; or (2) 2-h plasma glucose ≥ 200 mg/dL (11.1 mmol/L) during OGTT. The test should be performed as described by the WHO, using a glucose load containing the equivalent of 75-g anhydrous glucose dissolved in water; or (3) A1C ≥ 6.5% (48 mmol/mol). The test should be performed in a laboratory using a method that is NGSP certified and standardized to the DCCT assay; or (4) In a patient with classic symptoms of hyperglycemia or hyperglycemic crisis, a random plasma glucose ≥ 200 mg/dL (11.1 mmol/L).

### Statistical analysis

Data were statistically analyzed using statistical software SPSS 23.0. All measurement data were tested for normality (Kolmogorov–Smirnov test) and homogeneity of variance. Measurement data were expressed as mean ± standard deviation (SD), and the t-test was used for pairwise comparison of measurement data between each group. Counting data were compared using chi-square analysis. Spearman correlation was used to analyze the correlation between serum levels of Homer1, FGF23, SAH, and Hcy and Gensini score. Multivariate unconditional Logistic regression was used to analyze the risk factors of CHD. The level of statistical significance for all the above tests was defined at a probability value of less than 0.05 (*P* < 0.05).

## Results

### Demographic characteristics

CHD group: age ranges from 47 to 68 years, with average age of 52.78 ± 10.39 years old, including 76 males and 61 females. Control group: age ranges from 46–67 years, with average age of 52.66 ± 10.32 years old, including 78 males and 60 females. The age and gender of the two groups were comparable (*P* > 0.05). The BMI, TC, LDL-C, TG, and FBG levels of the SAP group, UAP group and AMI group were significantly higher than those of the control group, and the number of patients with smoking, alcohol consumption, hypertension, and diabetes history was significantly more than that of the control group, respectively (*P* < 0.05). The level of HDL-C of each subgroup was significantly lower than the control group (*P* < 0.05). The above indicators showed no significant difference among three subgroups (*P* > 0.05). See Table 1.

**Table 1:** Comparison of demographic characteristics between groups

Items	SAP group (n = 48)	UAP group (n = 46)	AMI group (n = 43)	Control group (n = 145)	Z/χ^2^	*P* value
Age (years)	48.4 ± 10.2	51.3 ± 10.7	53.8 ± 11.0	52.7 ± 10.3	2.589	0.053
Gender (male/female)	27/21	25/21	24/19	78/60	0.068	0.995
BMI (kg/m^2^)	24.61 ± 2.42*	24.99 ± 2.48*	25.59 ± 2.51*	22.13 ± 2.21	37.839	< 0.001
Smoking history (n)	7*	8*	9*	3	20.477	< 0.001
Alcohol consumption history (n)	7*	7*	10*	2	24.398	< 0.001
Hypertension history (n)	28*	28*	27*	0	124.706	< 0.001
Diabetes history (n)	16*	16*	15*	0	59.744	< 0.001
TC (mmol/L)	4.69 ± 0.78*	4.83 ± 0.81*	4.98 ± 0.83*	3.19 ± 0.79	99.996	< 0.001
HDL-C (mmol/L)	1.22 ± 0.21*	1.16 ± 0.19*	1.09 ± 0.18*	1.43 ± 0.22	42.797	< 0.001
LDL-C (mmol/L)	2.43 ± 0.46*	2.55 ± 0.47*	2.62 ± 0.49*	1.83 ± 0.51	48.594	< 0.001
TG (mmol/L)	1.68 ± 0.19*	1.73 ± 0.21*	1.79 ± 0.24*	1.33 ± 0.21	87.874	< 0.001
FBG (mmol/L)	6.57 ± 0.81*	6.63 ± 0.79*	6.89 ± 0.82*	5.18 ± 0.78	86.594	< 0.001
**Medication (n, %)**						
Aspirin	46	45	42	–	0.407	0.816
Statins	47	46	42	–	1.035	0.596
Nitrate	37	36	33		0.033	0.984
β-blocker	30	32	30	–	0.726	0.696
ACEI/ARB	24	24	23	–	0.114	0.945

Compared with the control group (*P < 0.05)

*SAP* stable angina pectoris, *UAP* unstable angina pectoris, *AMI* acute myocardial infarction, *BMI* body mass index, *TC* total cholesterol, *HDL-C* high-density lipoprotein cholesterol, *LDL-C* low-density lipoprotein cholesterol, *TG* triglyceride, *FBG* fasting blood-glucose, *ACEI* angiotensin-converting enzyme inhibitors, *ARB* angiotensin receptor blockers

### Comparison of Homer 1, SAH, Hcy, FGF 23 levels between different subgroups

The levels of serum SAH, Hcy, FGF23 and Homer1 in the SAP group, UAP group and AMI group were significantly higher than those in the control group (*P* < 0.05, respectively). Serum SAH, Hcy, Homer1, FGF23 levels in SAP group and UAP group were significantly lower than those in AMI group (*P* < 0.05, respectively), and serum SAH, Hcy, FGF23, Homer1 levels in SAP group were significantly lower than UAP group (*P* < 0.05, respectively). See Table 2.

**Table 2:** Comparison of serum levels of Homer1, SAH, Hcy and FGF23 among different subgroups

Items	SAP group (n = 48)	UAP group (n = 46)	AMI group (n = 43)	Control group (n = 145)	Z	*P* value
Homer1 (2^−△^Ct)	2.85 ± 0.19*	3.09 ± 0.16*^#^	3.37 ± 0.18*^#@^	2.38 ± 0.21	368.785	< 0.001
SAH (nmol/L)	55.11 ± 4.01*	58.83 ± 4.12*^#^	62.19 ± 4.39*^#@^	45.03 ± 2.18	432.896	< 0.001
Hcy (μmol/L)	15.34 ± 1.23*	16.01 ± 1.28*^#^	17.85 ± 1.34*^#@^	13.01 ± 1.04	231.217	< 0.001
FGF23 (pg/mL)	38.29 ± 3.22*	43.29 ± 3.19*^#^	49.83 ± 3.37*^#@^	31.92 ± 3.29	183.352	< 0.001

Compared with the control group (*P < 0.05), compared with the SAP group (^#^*P* < 0.05), Compared with the UAP group (^@^*P* < 0.05)

*SAP* stable angina pectoris, *UAP* unstable angina pectoris, *AMI* acute myocardial infarction, *SAH* S-adenosyl-l-homocysteine, *Hcy* homocysteine, *FGF* fibroblast growth factors

### Correlation analysis between serum Homer1, FGF23, SAH, and Hcy levels and Gensini score

Spearman correlation analysis showed that serum levels of HOMER1, FGF23, SAH and Hcy were positively correlated with Gensini score (r = 0.376, 0.623, 0.291, 0.372, *P* < 0.01, respectively). See Table 3.

**Table 3:** Univariate unconditional logistic regression analysis of coronary heart disease risk factors

Items	β value	s $\overline{x }$	Wald χ^2^	*P* value	OR (95%CI)
Smoking	1.379	0.509	5.773	0.013	3.978 (1.387–9.789)
Hypertension	1.932	0.861	5.003	0.023	6.832 (1.268–17.083)
Diabetes	2.398	0.951	6.409	0.012	11.238 (1.745–72.298)
Alcohol intake	0.622	0.177	12.583	0.000	1.812 (1.356–2.432)
Obesity	0.872	0.371	5.763	0.015	2.458 (1.176–4.936)
HDL-C	0.187	0.031	26.531	< 0.001	1.183 (1.001–1.429)
FGF23	0.519	0.071	53.891	< 0.001	1.698 (1.321–1.959)
SAH	0.192	0.075	7.217	0.006	1.201 (1.021–1.542)
Hcy	0.362	0.063	28.108	< 0.001	1.392 (1.246–1.647)
Homer1	1.191	0.417	0.679	0.005	1.376 (1.118–2.707)

*HDL-C* high-density lipoprotein cholesterol, *FGF* fibroblast growth factors, *SAH* S-adenosyl-l-homocysteine, *Hcy* homocysteine

### Univariate and multivariate unconditional Logistic regression analysis of risk factors

Age, gender and other influencing factors that may be potential correlated with the main risk factors for CVD have been adjusted before logistic regreesion. With the presence of CHD as the dependent variable, the above clinical data and serum Homer1, FGF23, SAH, and Hcy levels were used as independent variables. The results of multivariate logistic regression analysis showed that smoking, hypertension, diabetes, alcohol consumption, obesity, HDL-C, FGF23, SAH, Hcy, Homer1 were risk factors for coronary heart disease (Table 4).

**Table 4:** Multivariate unconditional Logistic regression analysis of coronary heart disease risk factors

Items	β value	s$\overline{x }$	Wald χ^2^	*P* value	OR (95%CI)
Smoking	1.379	0.509	5.773	0.013	3.978 (1.387–9.789)
Hypertension	1.932	0.861	5.003	0.023	6.832 (1.268–17.083)
Diabetes	2.398	0.951	6.409	0.012	11.238 (1.745–72.298)
Alcohol intake	0.622	0.177	12.583	< 0.001	1.812 (1.356–2.432)
Obesity	0.872	0.371	5.763	0.015	2.458 (1.176–4.936)
HDL-C	0.187	0.031	26.531	< 0.001	1.183 (1.001–1.429)
FGF23	0.519	0.071	53.891	< 0.001	1.698 (1.321–1.959)
SAH	0.192	0.075	7.217	0.006	1.201 (1.021–1.542)
Hcy	0.362	0.063	28.108	< 0.001	1.392 (1.246–1.647)
Homer1	1.191	0.417	0.679	0.005	1.376 (1.118–2.707)

*HDL-C* high-density lipoprotein cholesterol, *FGF* fibroblast growth factors, *SAH* S-adenosyl-l-homocysteine, *Hcy* homocysteine

## Discussion

Cardiovascular diseases (CVDs) cause about 31% of all deaths worldwide, with an estimate of over 17 million deaths in 2016 [18]. CHD accounts for the greatest proportion of CVDs [19]. The causes of heart attacks are usually the presence of a combination of risk factors, such as tobacco use, unhealthy diet and obesity, physical inactivity and harmful use of alcohol, hypertension, diabetes and hyperlipidaemia [18]. Despite great advances in recent years with the use of traditional risk factor control and treatment methods such as statins, a large number of patients with CHD are still at residual risk of cardiovascular events [19]. Therefore, actively looking for the risk factors of CHD and to intervene, is an important clinical problem to be solved urgently.

Hcy is a non-protein, sulfur-containing amino acid, and a metabolic intermediate in the methionine cycle [20]. Different studies have different explanations for the mechanism of Hcy leading to atherosclerosis (AS) [21–23]. A previous review of animal and human literature suggested that Hcy may also interfere with the expression of AS-related genes by interfering with DNA epigenetic phenotypic modifications, such as DNA methylation [21]. Another study showed that abnormal methylation of genes such as peroxisome proliferator-activated receptor α, apolipoprotein E (ApoE), and genomic DNA contributed to the development of Hcy-induced AS [22]. Also, another explanation was Homocysteine thialactone (a by-product of homocysteine auto-oxidation) could combine with native LDL to form oxidized LDL which was taken up by intimal macrophages to form foam cells which is the beginning of atheromatous plaques [23]. Zhang et al. [24] pointed out that plasma Hcy level has certain predictive value for CHD in elderly patients with hyperlipidemia, which is worthy of clinical application.

In recent years, the close relationship between HOMER and the onset and development of cardiovascular atherosclerosis has been gradually revealed [25–28]. Many studies have proved that HOMER stimulates the function of the phosphoprotein homolog 1(EVH1) domain with multiple receptors and multiple calcium-regulatory proteins in the body through vasodilation, including inositol 1,4,5-trisphosphate receptor (IP3R), metabolic glutamate receptor (mGluR), transient receptor potential channel (TRPC), and ryanodine receptors (RyRs) [25–27]. Jardin et al. [28] suggested that HOMER regulates the influx of calcium ions in human platelets by associating with calcium release-activated Orail 1 and stromal interaction molecule 1. All these evidences suggested that HOMER may play an important role in the development of atherosclerotic plaques. Jing et al. [29] found that the expression level of Homer1 in peripheral leukocytes of patients with CHD show an increasing trend. Another study suggested that the expression level of Homer1 in peripheral blood leukocytes of patients with CHD is closely related to the occurrence and development of cardiovascular diseases, and may have certain diagnostic value for CHD [30]. Moreover, Liu et al. [31] pointed out that the level of Homer1 in CHD patients is significantly different from that in healthy people, which is of great significance for the clinical diagnosis and disease evaluation and monitoring of CHD.

SAM is widely present in organisms in nature. It is a common physiologically active substance. It can play the role of transsulfuration, transmethylation, and transaminopropylation. It is also the precursor of taurine, coenzyme A, cysteine and other substances. Also, SAM plays an important role in liver disease, depression, Alzheimer's, arthritis and other diseases [32]. With the deepening of clinical research on methionine metabolism, methionine metabolites, SAH and S-adenosine methionine (SAM), have been found to play an important role in promoting the occurrence and development of AS. The mechanisms are as follows [32, 33]: (1) SAM is the methyl donor of numerous specific transmethylation reactions, which can be divided into two types: S type and R type, and S type SAM has biological activity. Under the action of methyltransferase, SAM transfers methyl to other macromolecular substances in vivo, such as proteins, and itself transformed into SAH, promoting the increase of SAH content. Adenosine is removed from SAH under the action of SAH hydrolase (SAHH) to form Hcy, and methionine is formed through methylation and transsulfur pathway, which is repeated to form methionine cycle. (2) In the process of methionine metabolism, Hcy can be reversed to SAH under the action of SAHH, so high methionine diet will increase the aggregation of Hcy, thus reverse conversion to SAH, and promote the increase of the intracellular level of SAH. The negative feedback of SAH inhibits DNA methyltransferase and reduces the methylation of SAM. With the gradual increase of SAM, it inhibits the methylation process and leads to the whole genomic DNA hypomethylation, which affects the expression of genes related to atherosclerotic lesions, thus promoting the occurrence and development of AS. Li et al. [34] found that compared with traditional serological indicators, plasma SAH level in CHD patients is significantly increased and the correlation between plasma SAH level and the degree of coronary artery disease was the highest. It may be a new biomarker for the clinical diagnosis of CHD.

FGF23 is synthesized in osteoblasts and plays an important role in the regulation of vitamin D level and parathyroid hormone hieroglyphic calcium and phosphorus metabolism. FGF23 increases renal phosphate excretion and limits the conversion of 25-hydroxyvitamin D to active 1, 25-dihydroxyvitamin D3. Low concentrations of 1, 25-dihydroxyvitamin D3 reduce phosphate absorption in the intestinal tract and promote secondary hyperparathyroidism, resulting in hyperphosphatemia, vitamin D deficiency, and increased parathyroid hormone levels. In addition, FGF23 is involved in regulating the activity of renal enzymes and reducing the activity of specific transporters, resulting in sodium—phosphate co-transport disorder and electrolyte metabolism imbalance [35]. Continuously elevated serum FGF23 level can lead to AS and vascular dysfunction, promote left ventricular hypertrophy, and cause irreversible effects on cardiac function, leading to cardiorenal syndrome [36]. Dai et al. [37] found that the expression of FGF23 in patients with heart failure was significantly higher than that in normal controls. The up-regulation of serum FGF23 expression in elderly CHD patients complicated with heart failure after PCI can be used as a reliable indicator of prognosis [38]. Increased serum FGF23 level and reduced serum Fetuin-A level are independent risk factors of major adverse cardiac events (MACE) after percutaneous coronary intervention (PCI) in ST-segment elevation myocardial infarction patients, which can be used as clinical indicators to predict MACE after PCI [39]. The results of this study showed that the BMI, smoking history, alcohol consumption history, hypertension, diabetes, TC, LDL-C, TG and FBG levels of SAP group, UAP group and AMI group were all higher than those of the control group, and the HDL-C level was lower than that of the control group. It suggested that BMI, smoking and alcohol consumption history, hypertension, diabetes, TC, LDL-C, TG and FBG levels were closely related to CHD. Furthermore, serum levels of SAH, Hcy, FGF 23, Homer 1 in SAP group, UAP group and AMI group were all higher than those in control group. SAH, Hcy, Homer 1, FGF 23 levels in SAP group and UAP group were all lower than those in AMI group. And the above indicators in SAP group were lower than those in UAP group. These results indicated that the serum levels of SAH, Hcy, FGF 23, Homer 1 were significantly increased in patients with CHD, and can reflect the severity of CHD to a certain extent.

Chi et al. [40] revealed that elevated Hcy can be used as a susceptibility factor of CHD and participate in the occurrence and development of the disease. Zhu et al. [41] pointed out that serum Hcy and HMGB1 were involved in the onset of CHD and were positively correlated with its severity. Zou et al. [42] suggested that serum SAH may be a better predictor of coronary heart disease than Hcy, and can predict the severity of coronary artery disease. Another study revealed that abnormal elevation of serum FGF23 was associated with major adverse cardiovascular events in patients with acute coronary syndrome after percutaneous coronary intervention, and could be used as a serum indicator for early evaluation of adverse cardiovascular outcomes in patients with acute coronary syndrome [43]. Tao et al. [44] found there was a significant correlation between FGF23 levels and TNF-α and IL-6 in inflammatory response indicators in patients with CHD. Results of Spearman correlation analysis in this study showed that serum levels of HOMER 1, FGF 23, SAH and Hcy were all positively correlated with Gensini score.

Multivariate unconditional Logistic regression analysis in this study showed that smoking, hypertension, diabetes, alcohol consumption, obesity, HDL-C, FGF23, SAH, Hcy, and HOMER1 were risk factors for CHD. It was suggested that CHD is closely related to smoking, hypertension, diabetes, alcohol consumption, obesity, HDL-C, FGF23, SAH, Hcy and HOMER1, and FGF23, SAH, Hcy and HOMER1 are new risk factors for CHD, which may have an important reference value for the clinical diagnosis of CHD. Tobacco contains nicotine, which can cause vasoconstriction, increase blood viscosity, accelerate the formation of thrombosis and plaque, and accelerate the rate of atherosclerosis. Therefore, smoking is closely related to CHD. The rise of systolic and diastolic blood pressure will increase the risk of CHD. If blood pressure can be effectively controlled, the risk of CHD can be reduced by 16%. Hypertension is closely related to the occurrence and development of coronary heart disease due to the continuous pressure of coronary artery, resulting in the damage of vascular endothelial cells and the rupture of plaque. Patients with CHD complicated with diabetes will increase the degree of diffuse coronary artery lesions to a certain extent. Therefore, diabetes may affect the prognosis of patients with CHD. Also, excessive alcohol consumption is associated with an increased risk of cardiovascular adverse events and cardiovascular mortality. People who are overweight or obese are more likely to develop metabolic syndrome, increase sympathetic excitability, reduce insulin sensitivity, increase the risk of CHD. Thus, obesity can be consider as a risk factor of CHD. Lipid metabolism disorder is the basis of atherosclerotic lesions. The lesions start from the intima. Complex carbohydrates and lipids accumulated first and then the plaques formed, leading to calcinosis and fibrous tissue hyperplasia, accompanied by degeneration and calcification of middle arterial layer, manifested as thickening and hardening of arterial wall and narrowing of arterial lumen. Therefore, when dyslipidemia accompanied by coronary artery stenosis, coronary atherosclerosis and coronary heart disease should be considered first. HDL-C, which is mainly composed of proteins and lipids, is an important serum protein involved in cholesterol reversal. It can transfer cholesterol and other substances from the vascular wall and other peripheral tissues to the liver, and promote cholesterol metabolism, reduce its accumulation in the vascular wall and other parts. In addition, it has functions like anti-inflammatory, anticoagulant, repairing damaged blood vessels, and antioxidant, so it plays an important role in coronary heart disease and other cardiovascular diseases. Duan et al. [45] indicated that the HDL-C level of patients with coronary stenosis ≥ 50% was significantly lower than that of patients with coronary stenosis < 50%. Low HDL-C is a risk factor for increased severity of coronary artery stenosis in patients with CHD [46]. FGF23 is closely related to the occurrence and development of CHD because it can accelerate the process of vascular lesions. SAH's inhibition of SAM-mediated responses has been identified as the mechanism leading to metabolic abnormalities. Hcy can reverse generate SAH, so it is speculated that the direct cause of Hcy's toxicity to vascular endothelial cells may be increased SAH, and vascular endothelial cell damage is the early pathological manifestation of AS. Elevated Hcy will damage the vascular wall, resulting in accumulation of lipid and other lesions, which will generate a large number of regenerative cells and induce coronary plaque. Meanwhile, Hcy can reduce HDL-C level by inhibiting apoA-1 protein expression. Homer1 plays an important role in the formation of atherosclerotic thrombosis by regulating the channel activity of calcium influx controlled by calcium pools, thereby regulating the cytoplasmic level, thereby regulating the concentration of Ca ions in the cytoplasm, and thereby regulating the activity of platelets.

There are some limitations of this study. First, it is a single center study, relatively small sample size may lead to bias. Secondly, the lack of mechanism exploration leads to the only clinical phenomenon presented in this study, which needs further research.

## Conclusion

In conclusion, the levels of FGF23, SAH, Hcy, and HOMER1 in CHD patients were significantly higher than those in normal control, and increased with the aggravation of the severity of the disease, which is of great significance for the clinical diagnosis and evaluation of the disease.

## Data Availability

The datasets used and/or analysed during the current study are available from the corresponding author upon reasonable request.

## Abbreviations

AMI: Acute myocardial infarction
AS: Atherosclerosis
BMI: Body mass index
CHD: Coronary heart disease
Ct: Cycle threshold
CVD: Cardiovascular diseases
DBP: Diastolic blood pressure
FBG: Fasting blood-glucose
FGF: Fibroblast growth factors
Hcy: Homocysteine
HDL-C: High density lipoprotein cholesterol
IP3R: Inositol 1,4,5-trisphosphate receptor
LDL-C: Low density lipoprotein cholesterol
mGluR: Metabolic glutamate receptor
RyRs: Ryanodine receptors
SAH: S-adenosyl-l-homocysteine
SAM: S-adenosine methionine
SBP: Systolic blood pressure
SD: Standard deviation
TC: Total cholesterol
TG: Triglyceride
TRPC: Transient receptor potential channel
UPA: Unstable angina pectoris
